# Prophylaxis, diagnosis and therapy of infections in patients undergoing high-dose chemotherapy and autologous haematopoietic stem cell transplantation. 2020 update of the recommendations of the Infectious Diseases Working Party (AGIHO) of the German Society of Hematology and Medical Oncology (DGHO)

**DOI:** 10.1007/s00277-020-04297-8

**Published:** 2020-10-20

**Authors:** Maximilian Christopeit, Martin Schmidt-Hieber, Rosanne Sprute, Dieter Buchheidt, Marcus Hentrich, Meinolf Karthaus, Olaf Penack, Markus Ruhnke, Florian Weissinger, Oliver A. Cornely, Georg Maschmeyer

**Affiliations:** 1 Department of Stem Cell Transplantation, University Medical Center Eppendorf, Hamburg, Germany; 2 Department of Hematology and Oncology, Carl-Thiem-Klinikum, Cottbus, Cottbus, Germany; 3 Cologne Excellence Cluster on Cellular Stress Responses in Aging-Associated Diseases (CECAD), University of Cologne, Cologne, Germany; 4 Department I of Internal Medicine, University Hospital of Cologne, University of Cologne, Cologne, Germany; 5 Partner Site Bonn-Cologne, German Centre for Infection Research, Cologne, Germany; 6 Department of Hematology and Oncology, Mannheim University Hospital, Heidelberg University, Mannheim, Germany; 7 Department of Medicine III—Hematology/Oncology, Red Cross Hospital, Munich, Germany; 8 Department of Internal Medicine, Hematology and Oncology, Klinikum Neuperlach, Städtisches Klinikum München, Munich, Germany; 9 Department of Internal Medicine, Division of Hematology and Oncology, Charité Universitätsmedizin Berlin, Campus Rudolf Virchow, Berlin, Germany; 10 Department of Hematology, Oncology and Palliative Medicine, Helios Hospital Aue, Aue, Germany; 11 Department of Internal Medicine, Hematology, Oncology, Stem Cell Transplantation and Palliative Medicine, Protestant Hospital of Bethel Foundation, Bielefeld, Germany; 12 Clinical Trials Centre Cologne (ZKS Köln), University of Cologne, Cologne, Germany; 13 Klinikum Ernst von Bergmann, Department of Hematology, Oncology and Palliative Care, Potsdam, Germany

**Keywords:** Autologous stem cell transplantation, Infection

## Abstract

To ensure the safety of high-dose chemotherapy and autologous stem cell transplantation (HDC/ASCT), evidence-based recommendations on infectious complications after HDC/ASCT are given. This guideline not only focuses on patients with haematological malignancies but also addresses the specifics of HDC/ASCT patients with solid tumours or autoimmune disorders. In addition to HBV and HCV, HEV screening is nowadays mandatory prior to ASCT. For patients with HBs antigen and/or anti-HBc antibody positivity, HBV nucleic acid testing is strongly recommended for 6 months after HDC/ASCT or for the duration of a respective maintenance therapy. Prevention of VZV reactivation by vaccination is strongly recommended. Cotrimoxazole for the prevention of *Pneumocystis jirovecii* is supported. Invasive fungal diseases are less frequent after HDC/ASCT, therefore, primary systemic antifungal prophylaxis is not recommended. Data do not support a benefit of protective room ventilation e.g. HEPA filtration. Thus, AGIHO only supports this technique with marginal strength. Fluoroquinolone prophylaxis is recommended to prevent bacterial infections, although a survival advantage has not been demonstrated.

## Introduction

High-dose chemotherapy (HDC) and autologous stem cell transplantation (ASCT) constitute a standard of care in the treatment of heamatologic malignancies, in particular, multiple myeloma [1], malignant lymphoma [2, 3] and acute myeloid leukaemia [4]. HDC and ASCT are also used in solid tumours such as neuroblastoma, sarcoma, germinal tumours [5] and autoimmune diseases e.g. multiple sclerosis [6], systemic sclerosis [7] and Crohn’s disease [8]. In total, the number of ASCT reported to the European Society for Blood and Marrow Transplantation in 2018 exceeds 27,750, including more than 560 for non-malignant disorders, mostly autoimmune diseases, and 1545 for solid tumours [9].

After HDC and ASCT, up to 90% of patients will experience infections, mostly presenting as fever of unknown origin (FUO) [10].

Due to indwelling venous catheters and toxicities of conditioning regimens, in particular, mucositis and gastrointestinal barrier impairment, both Gram-positive and Gram-negative bacteria may cause fever and infection after HDC/ASCT [11]. Bacterial pneumonia, colitis and bloodstream infection (BSI), including catheter-related BSI, are the prevailing documented infections in patients after HDC/ASCT [11, 12]. Invasive fungal disease and viral infections, ranging from herpes to hepatitis viruses, may occur as well, extending into the period after transplantation [13, 14].

## Methods

### Development of the guideline

These recommendations on prophylaxis, diagnosis and treatment of infectious complications after HDC/ASCT by the Infectious Diseases Working Party (AGIHO) of the German Society of Haematology and Medical Oncology (DGHO) are the fourth edition after 1999, 2003 and 2012 [15–17]. The expert panel assessed the recommendations in a stepwise consensus process, consisting of telephone and video conferences. All members of the AGIHO were invited to the consensus meetings. The guideline was approved by the AGIHO assembly on May 7, 2020.

This guidance document is embedded into other AGIHO recommendations on infection management in patients with haematological and oncological diseases [18–25]. It provides an evaluation of current evidence and the consensus interpretation of the authors. Recommendations are not mandatory and intend to assist physicians in decisions on individual patients. We regard correct dosing to be a responsibility of the prescribing physicians and do not mention this in this guideline.

Strength of recommendation and quality of evidence were graded according to the criteria applied by the European Society for Clinical Microbiology and Infectious Diseases (ESCMID) and the European Confederation of Medical Mycology (ECMM; Table 1) [26, 27].

**Table 1:** Definitions of strength of recommendation and quality of evidence

Category, grade	Definition
Strength of recommendation	
A	AGIHO strongly supports a recommendation for use
B	AGIHO moderately supports a recommendation for use
C	AGIHO marginally supports a recommendation for use
D	AGIHO supports a recommendation against use
Quality of evidence	
I	Evidence from at least 1 properly designed randomized, controlled trial
II	Evidence from at least 1 well-designed clinical trial, without randomization; from cohort or case-controlled analytic studies (preferably from > 1 centre); from multiple time series; or from dramatic results of uncontrolled experiments
IIr	meta-analysis or systematic review of RCT
IIt	transferred evidence, i.e. results from different patient cohorts or similar immune status situation
IIh	comparator group historical control
IIu	uncontrolled trials
IIa	published abstract, presented at an international symposium or meeting
III	Evidence from opinions of respected authorities, based on clinical experience, descriptive case studies

### Risk stratification

Both depth and duration of neutropenia have been associated with the risk of cancer patients to develop an infection that will take a more severe and complicated course [28]. AGIHO regards patients with expected neutropenia < 500/μl for at least 8 days to be at high risk and those with an expected duration of neutropenia of up to 7 days at standard risk for the development of an infection with a complicated course [18]. While it is generally presumed that patients after HDC/ASCT are amongst those with neutropenia lasting for 8 days or longer, thus are at high risk for complicated infection, data from prospective clinical trials show that the duration of neutropenia may often be in the standard risk range after HDC/ASCT [29]. However, profound, and sometimes long-lasting defects of components of the adaptive immune system are observed besides neutropenia after HDC/ASCT. Causes may lie in the nature of the underlying disease, timing and type of previous treatments, history of previous infection and specifics of the conditioning regimen e.g. nucleoside analogues or antilymphocyte globulins [6, 30–34]. In conclusion, AGIHO considers patients after HDC/ASCT at high risk for developing infections with a complicated course. Hereby, conditioning with carmustine, etoposide, cytarabine and melphalan (BEAM) prior to ASCT is associated with an increased risk for infections compared to conditioning with high-dose melphalan [35].

### Diagnostic procedures before the onset of fever or infection

Surveillance blood cultures in the absence of fever or other signs of infection are discouraged [36, 37]. Likewise, routine screening for invasive aspergillosis by serial determination of galactomannan antigen or 1,3-β-d-glucan is not recommended in these patients [38]. In individual patients at increased risk for invasive *Aspergillus* spp. infection, e.g. patients with previous aspergillosis who are without current systemic mould-active prophylaxis, twice-weekly galactomannan and/or 1,3-β-d-glucan surveillance may be considered. For more detailed information, refer to the separate guideline on diagnosis of fungal infection [20].

Screening for hepatitis B virus (HBV; anti-HBc antibodies, HBsAg, nucleic acid testing), hepatitis C virus (HCV; anti-HCV, nucleic acid testing), hepatitis E virus (HEV; nucleic acid testing) and human immunodeficiency virus (HIV; HIV1/2 antibodies, nucleic acid testing) is requested in all patients prior to release of the autologous graft [39, 40]. These screening tests should be repeated at least 30 days before HDC/ASCT. Monitoring of HBV viral load is strongly recommended in HBsAg and/or anti-HBc antibody positive patients for 6 months after HDC/ASCT, as immunosuppression can lead to viral reactivation and disease [41–45]. If maintenance therapy using rituximab, lenalidomide or bortezomib is administered, monitoring should be continued for 6 months following cessation of the respective maintenance therapy.

In patients with respiratory symptoms, screening for respiratory tract pathogens should include severe acute respiratory syndrome coronavirus 2 (SARS-CoV-2). HDC/ASCT may be deferred until the patient is asymptomatic [46, 47].

Diagnostic procedures recommended before onset of fever or infection are given in Table 2.

**Table 2:** Diagnostic procedures before the onset of fever or infection in HDC/ASCT recipients

Population	Intention	Intervention	SoR	QoE	References
Prior to HDC/ASCT	To detect HBV, HCV, HEV, HIV, and HDV status before release of autologous graft and and ≤ 30 days before HDC/ASCT	Screening for • HBV (anti-HBc antibodies, HBs antigen, nucleic acid testing) • HCV (anti-HCV, nucleic acid testing) • HEV (nucleic acid testing) • HIV (HIV1/2 antibodies, nucleic acid testing) • Anti-delta if HBsAg positive	A	III	RiliBÄK Dtsch Arztebl (2019) [40]
HBsAg and/or anti-HBc positive	To detect viral reactivation	HBV nucleic acid testing for ≥ 6 months after HDC/ASCT	A	IIu	Jun Hepatol Int (2017) [42] Kusumoto CID (2015) [44]
Unexplained elevated liver function tests	To detect viremia prior to HDC/ASCT	HEV nucleic acid testing	B	IIt	Von Felden J Hepatol (2019) [100] Furfaro BBMT (2020) [101]
CMV-seropositive	To detect CMV viremia and reduce CMV disease and CMV-related mortality	Routine screening for CMV viremia (CMV-PCR)	D	IIu	Marchesi Hematol Oncol (2018) [102] Kaya Transpl Proc (2017) [103] Massoud JCV (2017) [104] Piukovisc Ann Hematol (2017) [105]
HSV-seropositive	To detect HSV viremia and reduce HSV-related mortality	Routine screening for HSV viremia (HSV-PCR)	D	IIu	Inazawa JMV (2017) [76]
VZV-seropositive	To detect VZV viremia and reduce VZV-related mortality	Routine screening for VZV viremia (VZV-PCR)	D	III	No reference
Any	To reduce mortality and incidence of PTLD	Routine screening for EBV viremia (EBV-PCR)	D	IIu	Mehra CID (2019) [77] Inazawa JMV (2017) [76] Chiusolo JCI (2010) [106]
Any	To reduce the incidence of HHV-6 disease and infection-related mortality	Routine screening for HHV-6 (HHV-6-PCR)	D	IIu	Balsat J Infect (2019) [75] Inazawa JMV (2017) [76] Piukovics In Vivo (2014) [107]
Afebrile, neutropenic	To diagnose blood stream infection with the aim to reduce infection related mortality	Surveillance blood cultures	D	IIu	Ghazal Antimicrob Resist Infect Control (2014) [36]
Afebrile, neutropenic	To diagnose invasive aspergillosis	Surveillance serum galactomannan antigen	D	IItu	Duarte CID (2014) [108]
Afebrile, neutropenic	To diagnose invasive aspergillosis	Surveillance serum 1,3-β-d-glucan	D	IIt	Hammerström EJCMID (2015) [109] Cornely JAC (2017) [110]

### Diagnostic procedures in case of fever or infection

Thorough physical examination of a febrile neutropenic patient is mandatory. We strongly recommend two separate pairs of venous blood cultures in case of fever or other signs or symptoms of infection. In the presence of a central venous catheter (CVC), one of the two pairs should be obtained from the catheter. To increase the diagnostic yield of blood cultures, it is recommended with moderate strength to draw blood from each individual CVC lumen, or to obtain a third pair of blood cultures [48–50]. Determination of the differential time to positivity (DTTP) between blood cultures drawn from the CVC and a peripheral vein might be useful to identify the source of BSI [51]. DTTP of 2 h or more is suggestive of catheter-related BSI [52]. DTTP may be a useful tool to diagnose *Candida* spp. BSI, with a cut-off value of 6 h for *Candida glabrata* BSI and 2 h for other *Candida* spp. [53]. In contrast, DTTP has not proven useful in the diagnosis of *Staphylococcus aureus* BSI [54, 55]. In *S. aureus* or *Candida* spp. BSI CVC should be removed whenever possible, independent of the exact source of infection [24]. Chest X-rays are commonly discouraged to diagnose lung infection in tumour patients, since infiltrates are frequently invisible [56, 57]. High-resolution/multi-slice thoracic CT scan without contrast enhancement has a significantly higher sensitivity than chest X-ray and should be performed in patients with respiratory symptoms or persisting fever despite antimicrobial treatment over 72–96 h [22, 57]. Diagnostic bronchoscopy, bronchial or bronchoalveolar lavage for patients with pulmonary infiltrates (histology, cytology, culture, antigen testing, nucleic acid testing) should be applied whenever possible. Further diagnostics (e.g. abdominal or central nervous system imaging) might also be required, depending on symptoms, clinical signs and laboratory parameters. Signs of sepsis in the absence of fever as well as hypothermia should prompt diagnostic procedures as for a first fever, and empiric antibiotic treatment.

Diagnostic procedures recommended at onset of fever or infection are summarized in Table 3.

**Table 3:** Diagnostic procedures in fever or infection in HDC/ASCT recipients

Population	Intention	Intervention	SoR	QoE	References
First fever	To diagnose bloodstream infection	Analysis of two separate pairs of venous blood cultures (2× aerobic/2× anaerobic)	A	IIu	Lee JCM (2007) [48] Cockerill CID (2004) [49] Bouza CID (2007) [111]
Fever in presence of CVC	To diagnose bloodstream infection	Analysis of two separate pairs of venous blood cultures (2× aerobic/2× anaerobic), one of which is drawn from the catheter (total volume 40 ml)	A	IIu	Lee JCM (2007) [48] Cockerill CID (2004) [49] Planes EIMC (2016) [112]
Fever in presence of CVC	To increase diagnostic yield of blood cultures	Analysis of a blood sample from each lumen of the central venous catheter	B	IItu	Guembe CID (2010) [50] Herrera-Guerra AJIC (2015) [113]
Fever in presence of CVC	To increase diagnostic yield of blood cultures	Analysis of a third pair of blood cultures (total volume 60 ml)	B	IItu	Guembe CID (2010) [50]
Fever	To diagnose pneumonia	Chest X-ray in two projections	D	IIt	Gerritsen PLoSOne (2017) [57] Patsios Respir Med (2010) [56]
Respiratory symptoms	To diagnose pneumonia	Thoracic CT scan without contrast enhancement	A	IIu	Gerritsen PLoSOne (2017) [57]
Persistent fever for > 96 h despite broad antibacterial therapy	To diagnose pneumonia	Thoracic CT scan without contrast enhancement	A	IIu	Gerritsen PLoSOne (2017) [57]
Fever, neutropenia, and pulmonary infiltrates	To identify causative pathogens, for example *P. jirovecii*, Gram-negative bacteria, pneumococci, *Nocardia* spp*.*, *M. tuberculosis*, *Aspergillus* spp., *Mucorales* spp., respiratory viruses, incl. SARS-CoV-2	Bronchoscopy, bronchial or bronchoalveolar lavage (histology, cytology, culture, antigen testing, nucleic acid testing)	A	III	Marchesi AJH (2019) [114]

### Antimicrobial prophylaxis for patients during and after HDC/ASCT

#### Antibacterial prophylaxis

Prophylactic antimicrobial use can be considered in ASCT recipients during pre-engraftment when the duration of profound neutropenia (absolute neutrophil count < 100/μl) is expected to be at least 7 days. One placebo-controlled randomized trial has shown a reduction in the number of infections in patients, including those after HDC/ASCT, when using levofloxacin prophylaxis [58]. Moreover, a retrospective study with ASCT recipients has shown significant reduction of febrile neutropenia and BSI under prophylaxis with levofloxacin [10]. Of note, levofloxacin is not approved for antibacterial prophylaxis in Germany. An increasingly critical view of fluoroquinolone prophylaxis is explained by the absence of a survival benefit of fluoroquinolone prophylaxis in randomized clinical trials as well as by toxicities associated with fluoroquinolone use [58, 59]. Fluoroquinolone-resistant Enterobacteriaceae have been linked to community fluoroquinolone consumption. Prophylactic efficacy is reduced in neutropenic patients when the prevalence of fluoroquinolone resistance exceeds 20% in Gram-negative bacteria [60].

In summary, AGIHO recommends to critically weigh expected benefits of fluoroquinolone prophylaxis against the risks and consider local bacterial epidemiology and individual patient risk factors for fatal outcome of infections. AGIHO strongly recommends fluoroquinolone prophylaxis if the intention is to prevent bacterial infection. It cannot be expected to reduce risk of death with such prophylaxis [58].

*Clostridioides difficile* enteritis occurred in 3% vs. 8% after HDC/ASCT in a randomized comparison of fidaxomicin versus placebo [61]. Since the incidence of *C. difficile*–associated diarrhoea was low in both the placebo and in the fidaxomicin arm, AGIHO does not recommend prophylaxis of *C. difficile* enteritis after HDC/ASCT in the clinical routine.

#### Antifungal prophylaxis

Available data do not support the prophylactic use of antifungals to prevent invasive fungal disease (IFD). Those are rare events after HDC/ASCT and no reduction in mortality has been found in patients after HDC/ASCT [62, 63]. This is particularly true for mould-active antifungal agents. Yet, antifungal prophylaxis might be considered on a case-by-case basis if severe and long-lasting immunosuppression is expected.

The value of protective room ventilation such as high-efficiency particulate air (HEPA) filtration, positive pressure–directed airflow or laminar airflow is not well established for HDC/ASCT patients. During the last two decades, the use of air-filtered rooms has decreased as the incidence of filamentous fungal infections is extremely low in patients undergoing HDC/ASCT [64]. In a prospective clinical trial with 400 HDC/ASCT patients from nine transplant centres, no significant impact of HEPA filtration on the incidence of pneumonia, including IFD, or mortality rate was observed [65]. Thus, there are no data to mandate air-filtered rooms and AGIHO supports a recommendation of air-filtered rooms with marginal strength.

Prophylaxis against *Pneumocystis jirovecii* pneumonia (PJP) is efficacious with trimethoprim/sulfamethoxazole (TMP/SMX). A meta-analysis calculated an RR of 0.15 (95% CI 0.04–0.62) for an HIV-negative immunosuppressed individual to experience PJP when receiving prophylaxis with TMP/SMX, with a reduction of *P. jirovecii*–related mortality but no effect on all-cause mortality [66]. Reflecting mostly low quality of evidence due to bias and imprecision, AGIHO moderately supports a recommendation of TMP/SMX to prevent PJP after HDC/ASCT. If the administration of TMP/SMX is not feasible, alternatives are atovaquone, or pentamidine administered by inhalation or intravenously. These alternatives have not been studied in the HDC/ASCT setting and recommendations are transferred from other populations. The duration of *P. jirovecii*–directed prophylaxis should last for at least 3 months, preferably until the CD4+ T cell count stably exceeds 200/μl.

#### Antiviral prophylaxis

There are no large randomized controlled trials that evaluate antiviral drugs to prevent herpes simplex virus (HSV) and varicella zoster virus (VZV) disease after HDC/ASCT. Newer studies—including recently published placebo-controlled vaccination trials—showed that HSV infection, in particular, gingivostomatitis, and herpes zoster are still of concern in these patients [67–69]. Noteworthy, herpes zoster also frequently occurred beyond 6 months post-transplant, particularly in unvaccinated patients [69]. Antiviral drug use was reported in ~ 90% in this trial, with a duration of > 6 months in ~ 40% [69]. Several studies, including placebo-controlled studies and meta-analyses (but not focused on ASCT patients) showed that acyclovir is useful to prevent and treat HSV and VZV diseases [70, 71]. Thus, we strongly recommend acyclovir prophylaxis for at least 6 months, in particular, if CD34-selected grafts are used [34, 72]. Inactivated VZV vaccine is strongly recommended for all seropositive HDC/ASCT patients [69].

Cytomegalovirus (CMV) infection (reactivation) has been reported in some patients of mainly retrospective analyses focused to patients with fever after HDC/ASCT [73, 74]. However, CMV disease, i.e. organ involvement, is very rare in this setting [74]. In summary, CMV is no major concern in patients after HDC/ASCT. AGIHO supports a recommendation against the use of CMV prophylaxis.

Human herpes virus 6 (HHV-6) and Epstein-Barr virus (EBV) infections are infrequent after HDC/ASCT and routine prophylaxis is not recommended [75, 76]. However, EBV reactivation has been reported at a high frequency in patients with multiple sclerosis undergoing ASCT [77]. Thus, nucleic acid–based screening for EBV viremia at follow-up presentations and preemptive treatment with rituximab in the case of EBV infection should be considered in these patients.

Reactivation of viral hepatitis in patients after HDC/ASCT with previous or ongoing chronic HBV infection i.e. HBsAg and/or anti-HBc positivity is associated with considerable morbidity and mortality. Reactivation has long been prevented using lamivudine. Lamivudine resistance can reach more than 70% in primary treatment of viral hepatitis B after long-time therapy [78]. Here, viral clearance and HBeAg status, amongst others, are important risk factors for the development of resistance. Today, tenofovir and entecavir are antiviral substances with resistance rates of 1–5%. Their prophylactic administration is safe even in combination with most of the conditioning regimens [79, 80]. AGIHO strongly recommends the use of either tenofovir or entecavir in patients with HBsAg- and/or anti-HBc positivity. Patients should regularly be monitored for reactivation despite antiviral prophylaxis by HBV DNA measurements. In addition to patients after HDC/ASCT, patients under steroid medication and patients after the use of anti-CD20-antibodies, e.g. during maintenance therapy after HDC/ASCT, are at high risk for HBV reactivation [81].

Notably, the presence of anti-HBc IgM indicates acute infection and should trigger HBV DNA PCR. In acute HBV infection, clinical priority will frequently be to treat hepatitis before continuing antineoplastic treatment. For negative HBV DNA PCR, a combination of HBsAg negativity, anti-HBc positivity, anti-HBs negativity most frequently is a sign of resolved infection. As it can also mirror low-level chronic infection or resolving acute infection, AGIHO recommends repeating such test to rule out a false-positive result for anti-HBc. Likewise, virustatic prophylaxis as outlined before and close monitoring of HBV DNA load is recommended if this specific constellation is repeatedly documented.

AGIHO as well as the German standing committee on vaccination recently published separate guidelines on anti-infective vaccination measures against vaccine-preventable diseases with an own section on the situation after HDC/ASCT [82, 83].

Recommendations on antimicrobial prophylaxis are summarized in Table 4.

**Table 4:** Antiinfective prophylaxis in HDC/ASCT recipients

Population	Intention	Intervention	SoR	QoE	References
Any	To prevent infection	Any fluoroquinolone	A	I	Bucaneve NEJM (2005) [58]
Any	To reduce mortality	Any fluoroquinolone	C	I	Bucaneve NEJM (2005) [58] Signorelli TID (2020) [10]
Any	To prevent IFD	Primary prophylaxis with mould active antifungal	D	II	Van Burik CID (2004) [63]
Any	To prevent invasive candidiasis	Primary prophylaxis with fluconazole 400 mg/d	D	I	Van Burik CID (2004) [63] Rotstein CID (1999) [62] Goodman NEJM (1992) [115]
Patients with previous IFD	To prevent recurrence of IFD	Secondary prophylaxis with the last successfully used antifungal	A	IIt	Cornely Mycoses (2019) [116] Sun BBMT (2015) [117]
Any	To prevent invasive fungal infection	Protective room ventilation e.g. HEPA filtration	C	IIu	Vokurka JCN (2013) [65] Dadd J Ped Oncol Nurs (2003) [118]
Any	To prevent PJP	Cotrimoxazole	B	IIr	Stern Cochrane (2014) [66]
Any	To prevent HSV reactivation	Acyclovir/valacyclovir	A	IIt	Kawamura IJH (2015) [70] Glenny Cochrane Database (2009) [119]
CD34-selected or enriched transplant	To prevent HSV reactivation	Acyclovir/valacyclovir	A	IIu	Van Laar JAMA (2014) [34] Lin Int J Hematol (2008) [72]
Seropositive for VZV	To prevent VZV reactivation	Vaccination	A	I	Winston Lancet (2018) [69] Dagnew Lancet ID (2019) [120]
Any	To prevent VZV reactivation	Acyclovir	A	IIu	Winston Lancet (2018) [69] Kawamura IJH (2015) [70] Sahoo BBMT (2017) [121] Seo Antivir Res (2017) [122]
CD34-selected or enriched transplant	To prevent VZV reactivation	Acyclovir	A	IIu	Crippa BBMT (2002) [123]
Any	To reduce CMV infection/disease	CMV prophylaxis (e.g. with foscarnet or acyclovir)	D	IIu	Boeckh JID (1995) [124]
HBs antigen or anti-HBc antibody positive	To prevent hepatitis B reactivation	Tenofovir or entecavir	A	IIt	Huang JCO (2013) [125] Lau Gastroenterol (2003) [126]

### Empiric antimicrobial therapy of fever of unknown origin

Empiric treatment of FUO during neutropenia after HDC/ASCT should follow recently published guidelines on the management of high-risk febrile neutropenia [18]. In summary, AGIHO strongly recommends to use single-agent broad-spectrum *Pseudomonas*-active antibiotics such as piperacillin/tazobactam, ceftazidime, cefepime, meropenem or imipenem/cilastatin as first line antibiotic therapy [18, 84]. As there is no systematic data on the empiric use of doripenem [85], ceftazidime-avibactam [86], ceftolozane-tazobactam [87] or cefozopran [88] in this setting, they are not discussed here. The upfront routine addition of tigecycline to first-line beta-lactam with anti-pseudomonal activity did not reduce the mortality rate and is therefore not recommended outside settings of high-level multidrug resistance [84]. Upfront addition of antibiotics with activity against Gram-positive bacteria such as glycopeptides (e.g. vancomycin, teicoplanin) [89], oxazolidinones (e.g. linezolid) or cyclic lipopeptides (e.g. daptomycin) has either shown no benefit or has not been studied properly, thus, we do not recommend their use. In patients with known colonization by VRE, addition of linezolid was not beneficial [90, 91]. The administration of antimicrobial agents active against MRSA or ESBL-producing Gram-negative bacteria is recommended with marginal strength in case of colonization with these pathogens. The addition of an aminoglycoside in clinically stable patients in first-line therapy is not recommended [92–94]. However, in case of clinical instability, their addition is moderately recommended as might the addition of glycopeptides. Changing the first-line antibiotic regimen in a clinically stable patient who is still febrile after > 96 h is not generally recommended [18, 95, 96].

Empiric use of antifungals during first-line therapy in a clinically stable patient with FUO after HDC/ASCT is not recommended [97–99]. However, second-line addition of a mould-active antifungal is recommended with marginal strength for patients with persisting fever for at least 96 h receiving a broad-spectrum first-line antibacterial therapy who had not received prior antifungal prophylaxis and who are expected to experience neutropenia for more than 7 days [97–99]. This is not recommended if a mould-active antifungal prophylaxis had been administered prior to signs of infection or fever. For second-line empiric antifungal therapy, caspofungin or liposomal amphotericin B are preferred, while conventional amphotericin B deoxycholate is not recommended due to an unfavourable toxicity profile.

Recommendations on empiric antimicrobial therapy are summarized in Table 5.

**Table 5:** Empirical antimicrobial therapy in HDC/ASCT recipients

Population	Intention	Intervention	SoR	QoE	References
Patients at onset of fever	To treat presumed underlying infection	Broad-spectrum antibiotics (piperacillin/tazobactam, ceftazidime, cefepime, meropenem, imipenem/cilastatin)	A	I	Bucaneve JCO (2014) [84] Reich BJH (2005) [127] Horita CMI (2017) [128] Harter BMT (2006) [129]
Patients at onset of fever, clinically stable	To treat presumed underlying infection	Add aminoglycoside	D	I	Del Favero CID (2001) [92]
Patients at fever onset, hospital with high rates of multidrug resistant bacteria	To treat presumed underlying infection	Add antibiotics as appropriate (e.g. novel combinations of cephalosporins and betalactamase inhibitors, siderophore cephalosporins, tigecycline)	A	I	Bucaneve JCO (2014) [84]
Patients at fever onset or with persisting fever	To treat presumed underlying infection	Add glycopeptide or oxazolidinone (e.g. linezolid)	D	I	Cometta CID (2003) [96] Lisboa IJID (2015) [90]
Patients with fever persisting > 96 h, clinically stable	To treat presumed underlying infection	Continue first line antibiotic treatment	A	I	Bow CID (2006) [130] Cometta CID (2003) [96]
Patients with a first fever	To treat presumed underlying infection	Add antifungal	D	IIt	Maschmeyer EJCMID (2013) [131]
Patients with fever persisting > 96 h, clinically stable	To treat presumed underlying infection	Add liposomal amphotericin B or caspofungin	C	IIt	Walsh NEJM (2004) [132] Walsh NEJM 1999 [133]

### Clinically documented infections

Recommendations on management of pulmonary infiltrates [22], gastrointestinal and perineal infections [23], central nervous system infection [25], central venous catheter–related infection [24] and invasive fungal infections [21] can be found in the respective guidelines of the Infectious Diseases Working Party (AGIHO) of the German Society of Haematology and Medical Oncology (DGHO).
